# Can the systemic inflammation score be used to predict prognosis in gastric cancer patients undergoing surgery? A systematic review and meta-analysis

**DOI:** 10.3389/fsurg.2022.971326

**Published:** 2022-09-19

**Authors:** Shuai Liu, Xiaowei Yu, Feifei Ye, Liangxian Jiang

**Affiliations:** ^1^ Department of Gastrointestinal Surgery, Taizhou Hospital of Zhejiang Province, Taizhou, China; ^2^ Department of Day Care Ward, Taizhou Hospital of Zhejiang Province, Taizhou, China

**Keywords:** gastric cancer, inflammation, prognosis, survival, surgery

## Abstract

**Background:**

Inflammatory markers are being increasingly used to predict the prognosis of cancer patients. We hereby conducted the first meta-analysis assessing the association between systemic inflammation score (SIS) and prognosis of gastric cancer patients undergoing surgical intervention.

**Methods:**

A literature search was carried out on PubMed, CENTRAL, Scopus, and Embase up to 3rd June 2022 for relevant studies. Adjusted data reported as hazard ratios (HR) was combined in a random-effects model.

**Results:**

A total of seven studies with 5,338 patients could be included. All studies were from either China or Japan and published in the last four years. Meta-analysis showed that higher SIS scores (1 or 2) were significant predictors of poor overall survival (OS) in gastric cancer patients (HR: 1.25 95% CI: 1.05, 1.49, *I*^2 ^= 11%). Similarly, the meta-analysis demonstrated that an SIS score of 2 was associated with poor OS as compared to scores of 0/1 (HR: 2.53 95% CI: 1.30, 4.89, *I*^2 ^= 45%). Data on disease-free survival (DFS) was scarce to draw conclusions.

**Conclusion:**

The SIS score can be a simple and useful tool to predict OS in gastric cancer patients undergoing surgery. Data on DFS is scarce and conflicting. Future studies should report using standard reference groups and provide data on DFS to enhance current evidence.

**Systematic Review Registration:** https://www.crd.york.ac.uk/prospero/#searchadvanced, identifier: CRD42022335548.

## Introduction

Gastric cancer is a serious health problem worldwide owing to its high prevalence and dismissal survival rates (1). While data indicates that the total burden of gastric cancer may be on a decline due to a reduced number of smokers and a decrease in *Helicobacter pylori* prevalence, the burden of the disease is large enough to concern amongst healthcare professionals (2). Indeed, gastric cancer is the fourth commonest malignancy and the third leading cause of cancer-related death in the world (3). Many patients with gastric cancer are diagnosed in advanced stage and metastasis or recurrence are important contributors to poor prognosis in such patients. At present, the American Joint Committee on Cancer (AJCC) proposed tumor-node-metastasis (TNM) classification is one of the commonest prognostic models used worldwide (4). However, precise prediction is still difficult and there is a need for simple and easily measurable prognostic factors for routine clinical practice.

Cancer inflammation has been explored recently to estimate patient prognosis. Studies have shown that cancer-related inflammation can cause DNA damage, mutations, proliferation of blood vessels, as well as growth, invasion, and metastasis of cancer cells (5, 6). Furthermore, the microenvironment surrounding the tumor is not only influenced by cancer itself but also depends on the host inflammatory response (7). In this context, several blood-based inflammatory markers like the neutrophil, lymphocyte, monocyte, and platelet count; albumin level; alcohol dehydrogenase; C-reactive protein (CRP), and combinations like neutrophil-to-lymphocyte ratio (NLR), lymphocyte-to-monocyte ratio (LMR), and platelet-to-lymphocyte ratio (PLR) have been used to predict outcomes in patients with cancer (8–10).

One such marker which is recently being used is the systemic inflammation score (SIS), which is based on the combination of serum albumin and LMR scores in the perioperative period. The SIS has been demonstrated to be of good prognostic ability in patients with lung, colorectal and esophageal cancer (11–13). The strength of the score lies in the combined use of albumin which is a nutritional marker and LMR which is an inflammatory marker (13). The SIS score ranges from 0 to 2. Generally, patients with albumin levels of ≥4.0 g/dl and LMR ≥ 4.44 are classified with a score of 0, those with either albumin <4.0 g/dl or LMR < 4.44 are given a score of 1, and those with albumin <4.0 g/dl and LMR < 4.44 were given a score of 2 (11, 12). Studies have shown that higher the score poorer is the cancer prognosis (11–13). In recent times, several studies have also reported the prognostic ability of SIS for gastric cancer patients but with varying results. At this point, it is unclear if SIS can be clinically used to predict outcomes of gastric cancer. Hence, we conducted the first systematic review and meta-analysis to assess if SIS is associated with outcomes in gastric cancer patients undergoing surgical intervention.

## Material and methods

### Inclusion and exclusion criteria

Our review was prospectively registered on PROSPERO (No. CRD42022335548) and reported based on the recommendations of the PRISMA statement (14). We included all types of studies reporting the association between perioperative SIS and outcomes of gastric cancer patients undergoing surgical intervention. The outcomes were to be either overall survival (OS) or disease-free survival (DFS) reported as adjusted ratios. We excluded studies not reporting separate outcomes for gastric cancer or not including those with surgical intervention. Furthermore, case reports, reviews, and editorials were also not included. If there were two or more studies with overlapping datasets, the largest study was included in the review.

We looked across the databases of PubMed, CENTRAL, Scopus, and Embase up to 3rd June 2022 for eligible English-language studies. We used a combination of free-text and MeSH search terms namely, “gastric cancer”, “gastric carcinoma”, “gastric malignancy”, “systemic inflammation score”, “survival”; and “cancer” for the literature search. The search strings used for all databases are presented in Supplementary Table S1. The first set of search outcomes was checked by the article titles and abstracts to weed out non-relevant articles. Two reviewers then selected studies for full-text analysis and cross-checked them against the eligibility criteria mentioned earlier. Only studies fulfilling all criteria were included in the review. All disagreements between the reviewers were solved in consultation with another reviewer. The references of included studies were also cross-checked for any missed articles.

Details of the first author, year of publication, location of the study, study population, tumor stage, timing of measurement of SIS, tumor stage, sample size, age, male gender, histology, tumor location, surgery type, adjuvant chemotherapy, follow-up and adjusted ratios of the outcomes were extracted by two reviewers using a previously prepared word document. The outcomes of the review were OS and DFS.

Quality assessment of the studies was also carried out by two authors of the review using the Newcastle Ottawa Scale (NOS) for observational studies (15). The scale has three components: study population, comparability, and outcomes with each component awarded points based on the relevant questions. The highest score achievable is nine.

### Statistical analysis

All of the included studies reported OS and DFS as hazard ratios (HR) with 95% confidence intervals (CI). These were combined to estimate the pooled effect size as HR in a random-effects model. We assessed inter-study heterogeneity using the *I*^2^ statistic. *I*^2 ^= 25%–50% meant low, 50%–75% meant medium, and more than 75% meant substantial heterogeneity. As the total number of studies was few, we could not use funnel plots to look for publication bias. However, a sensitivity analysis was performed and individual studies were excluded from the meta-analysis to look out for any change in the significance of the results. We also assessed the relationship of high SIS (scores 1–2) and various clinicopathological features of gastric cancer by calculating odds ratios and 95% CI. The factors analyzed were age (≥70 years vs. <70 years), gender (male vs. female), tumor size (≥5 cm vs. <5 cm), location (lower third vs. other sites), differentiation (poorly differentiated vs. well differentiated), lymph node metastasis (present vs. absent), tumor stage (stage III vs. stage I–II). All analyses were done on “Review Manager” [RevMan, version 5.3; Nordic Cochrane Centre (Cochrane Collaboration), Copenhagen, Denmark; 2014]. Quantitative analysis was only performed if at least 3 studies were reporting numerical data otherwise, a qualitative assessment was carried out.

## Results

**Figure 1:**
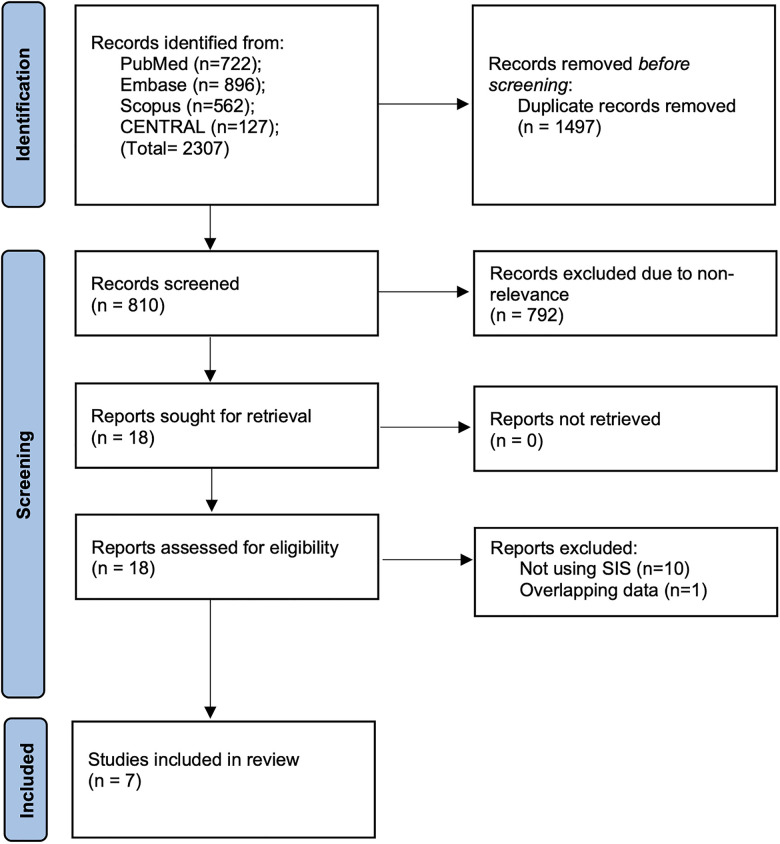
PRISMA study flowchart denoting number of articles at every stage of the inclusion process.

The outcomes of the literature search at every stage are presented in detail in Figure 1. Out of 810 unique articles screened, 18 were assessed by their full texts. We excluded 11 studies not fulfilling the inclusion criteria and managed to include seven studies in this review (16–22).

The baseline details extracted from the studies are presented in Table 1. All seven studies were either from China or Japan and published in the past four years. Most studies including patients with stage I–III cancer. The definition of SIS was the same across all studies except one. Inagaki et al. (20) used the LMR value of 3.4 instead of 4.44 which was common in the remaining studies [Value of 3.4 indicates the modified SIS (mSIS)]. Albumin values were the same across the included studies. SIS was calculated based on presurgical blood investigation in all studies except for Hara et al. (19) which used 1-month post-surgical values. The total number of patients analyzed was 5,338. The median age was >55 in all studies. Male patients predominated compared to female patients across the included studies. Five studies included patients only with adenocarcinoma. The percentage of poorly differentiated tumors ranged from 22.2% to 70.7%. Patients undergoing total gastrectomy varied from 27.2% to 51.1%. Adjuvant chemotherapy was used in 24% to 77.5% of patients. Median follow-up ranged from 35 to 101 months. All were good quality studies with the NOS score of 7–8.

**Table 1:** Details of included studies.

Study	Location	Study population	Tumor stage	SIS definition	Timing of SIS	Sample size	Age (years)	Male gender (%)	Adeno-carcinoma (%)	Poorly differentiated (%)	Location U/M/L/Mix	Total gastrectomy (%)	AC (%)	Median follow-up (months)	NOS score
He 2022 (22)	China	Early stage gastric cancer	I–II	0: Al ≥ 40 g/dl / LMR ≥ 4.44 1: either Al ≥ 40 g/dl or LMR ≥ 4.44 2: Al < 4 0 g/dl / LMR < 4.44	Pre-surgery	358	61	79.3	74	70.7	170/44/144/0	NR	44.4	101	8
Lin 2021 (21)	China	Primary gastric adenocarcinoma	I–III	0: Al ≥ 40 g/dl / LMR ≥ 4.44 1: either Al ≥ 40 g/dl or LMR ≥ 4.44 2: Al < 4 0 g/dl / LMR < 4.44	Pre-surgery	2180	NR	74.8	100	22.2	503/461/935/281	51.1	53.4	54	8
Inagaki 2021 (20)	Japan	Gastric adenocarcinoma	I–III	0: Al ≥ 40 g/dl / LMR ≥ 3.4 1: either Al ≥ 40 g/dl or LMR ≥ 3.4 2: Al < 4 0 g/dl / LMR < 3.4	Pre-surgery	1764	67.3	70.3	100	48.8	NR/NR/622/NR	27.2	27.5	62.9	8
Hara 2020 (19)	Japan	Advanced gastric cancer	II–III	0: Al ≥ 40 g/dl / LMR ≥ 4.44 1: either Al ≥ 40 g/dl or LMR ≥ 4.44 2: Al < 4 0 g/dl / LMR < 4.44	1 month post-surgery	160	67	63.7	100	61.9	NR	47.5	77.5	NR	7
Chen 2020 (18)	China	Gastric adenocarcinoma	I–IV	0: Al ≥ 40 g/dl / LMR ≥ 4.44 1: either Al ≥ 40 g/dl or LMR ≥ 4.44 2: Al < 4 0 g/dl / LMR < 4.44	Pre-surgery	358	59	62.3	100	63.1	60/34/165/99	45	24	35	7
Ma 2019 (17)	China	Gastric adenocarcinoma	I–III	0: Al ≥ 40 g/dl / LMR ≥ 4.44 1: either Al ≥ 40 g/dl or LMR ≥ 4.44 2: Al < 4 0 g/dl / LMR < 4.44	Pre-surgery	331	55.3	68.9	100	48.3	52/279	NR	NR	61.2	8
Sato 2018 (16)	Japan	pT2–4 gastric cancer	I–III	0: Al ≥ 40 g/dl / LMR ≥ 4.44 1: either Al ≥ 40 g/dl or LMR ≥ 4.44 2: Al < 4 0 g/dl / LMR < 4.44	Pre-surgery	187	69	78.1	NR	62	60/63/61/3	37	47	45.2	8

AC, adjuvant chemotherapy; Al, albumin; LMR, lymphocyte-monocyte ratio; SIS, systemic inflammation score; NOS, Newcastle Ottawa scale; U, upper; M, middle; L, lower; NR, not reported.

### SIS and clinicopathological features

**Figure 2:**
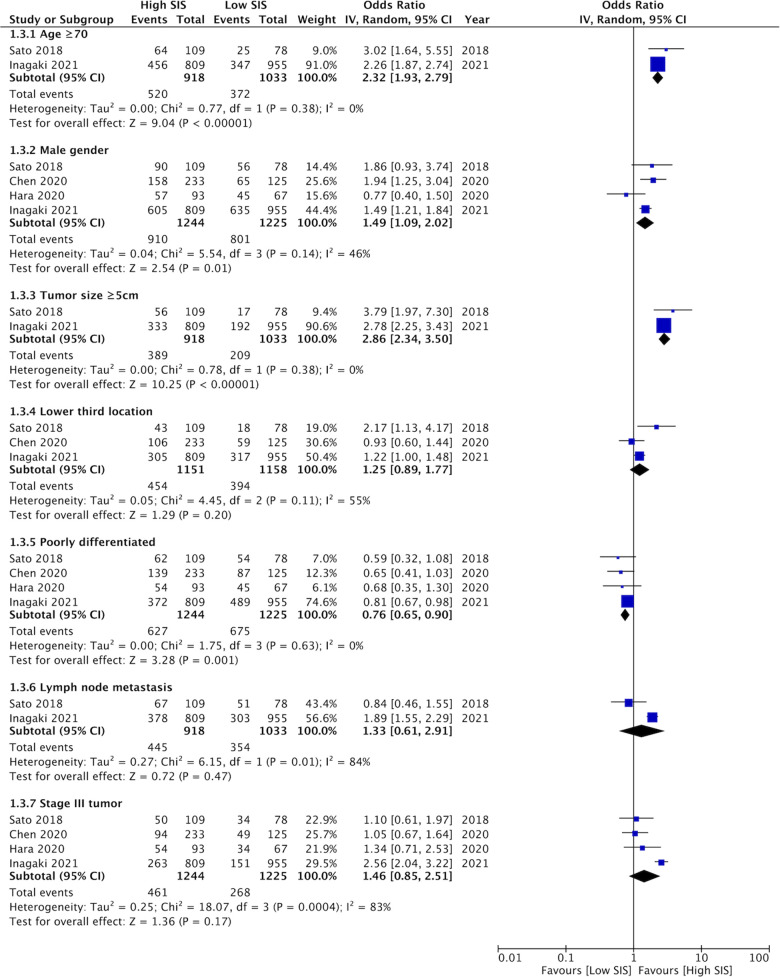
Meta-analysis assessing the relationship between high SIS and clinicopathological features of gastric cancer namely, age (≥70 years vs. <70 years), gender (male vs. female), tumor size (≥5 cm vs. <5 cm), location (lower third vs. other sites), differentiation (poorly differentiated vs. well differentiated), lymph node metastasis (present vs. absent), and tumor stage (stage III vs. stage I–II). Blue boxes indicate the point estimates of individual studies and horizontal lines denote the confidence intervals. Black diamond indicates the total effect size.

Details of clinicopathological characteristics of gastric cancer for different SIS scores were reported by a limited number of studies. Our meta-analysis of 2–4 studies showed that high SIS scores was associated with older age (≥70 years) (OR: 2.32 95% CI: 1.93, 2.79, *I*^2 ^= 0%), male gender (OR: 1.49 95% CI: 1.09, 2.02, *I*^2 ^= 46%), and tumor size ≥5 cm (OR: 2.86 95% CI: 2.34, 3.50, *I*^2 ^= 0%); while low SIS was associated with poorly differentiated gastric cancer (OR: 0.76 95% CI: 0.65, 0.90 *I*^2 ^= 0%) (Figure 2). SIS did not correlate with lower third tumor location (OR: 1.25 95% CI: 0.89, 1.77, *I*^2 ^= 55%), lymph node metastasis (OR: 1.33 95% CI: 0.61, 2.91, *I*^2 ^= 84%), or tumor stage III (OR: 1.46 95% CI: 0.85, 2.51, *I*^2 ^= 83%).

### OS

**Figure 3:**
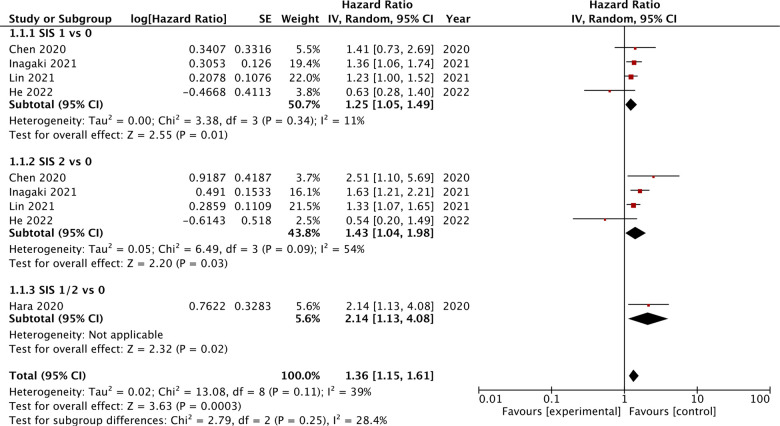
Meta-analysis of the association between SIS score and OS in gastric cancer patients with subgroup analysis based on different reference SIS score (1 vs. 0, 2 vs. 0, or 1–2 vs. 0). Blue boxes indicate the point estimates of individual studies and horizontal lines denote the confidence intervals. Black diamond indicates the total effect size.

**Figure 4:**
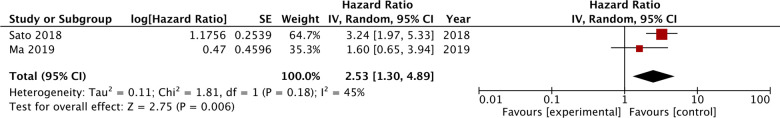
Meta-analysis of the association between SIS score of 2 vs. reference value of 0/1 for OS in gastric cancer patients. Blue boxes indicate the point estimates of individual studies and horizontal lines denote the confidence intervals. Black diamond indicates the total effect size.

Four studies compared the prognostic ability of SIS scores 1 and 2 vs. 0 for OS. On meta-analysis, we noted that SIS scores of 1 (HR: 1.25 95% CI: 1.05, 1.49, *I*^2 ^= 11%) and 2 (HR: 1.43 95% CI: 1.04, 1.98, *I*^2 ^= 54%) were associated with statistically significant poor OS as compared to those with SIS score of 0 (Figure 3). Hara et al. (19) comparing SIS scores of 1/2 vs. 0 noted a significant association between higher scores and poor OS (HR: 2.14 95% CI: 1.13, 4.08). Combining all the above data we noted that higher SIS scores (1 or 2) were significant predictors of poor OS in gastric cancer patients (HR: 1.25 95% CI: 1.05, 1.49, *I*^2 ^= 11%) (Figure 3). On sensitivity analysis for the entire data, we noted that the final result was still statistically significant on the exclusion of any of the included data. Two studies compared the SIS score of 2 vs. 0/1. The meta-analysis demonstrated that an SIS score of 2 was associated with poor OS as compared to scores of 0/1 (HR: 2.53 95% CI: 1.30, 4.89, *I*^2 ^= 45%) (Figure 4).

### DFS

Data on DFS was reported by a limited number of studies with varying comparisons which precluded a meta-analysis. Hara et al. (19) noted that the SIS score of 1/2 vs. 0 was not predictive of DFS (HR: 1.814 95% CI: 0.993–3.315) in gastric cancer patients. Sato et al. (16) also have shown that the SIS score of 2 vs. 0/1 was not associated with poor DFS (HR: 1.58 95% CI: 0.78–3.07). However, Ma et al. (17) noted that an SIS score of 2 was significantly associated with poor DFS as compared to those with scores of 0/1.

## Discussion

Most clinicians classify gastric cancer based on the pathological TNM staging system to determine patient prognosis and the need for additional therapies (23). Nevertheless, it is not uncommon to find varying OS and DFS in gastric cancer patients with the same tumor stage (24). This is somewhat attributable to the limitation of TNM staging which is based on the biological characteristics of the malignancy and does not incorporate the host and tumor inflammatory response (7). Indeed, the interaction between cancer and inflammation is quite complex. Systemic inflammatory changes can either herald the development of cancer or the malignancy itself can lead to a tumor-promoting inflammatory environment. Cancer cells produce pro-mediators like interleukins, transforming growth factor-*β*, macrophage migratory inhibitory factors, proteases, and eicosanoids which creates a pro-inflammatory environment that aids in the multiplication and survival of cancer cells, promotes angiogenesis and metastasis, attenuates adaptive immunity, and also changes the tumor response to hormones and chemotherapy (25). One way of estimating the inflammatory status of an individual is by measuring the cellular along with non-cellular elements of blood which are reflective of the immune and inflammatory status of the body. Kim et al. (8) in a meta-analysis have shown that NLR, CRP, and Glasgow prognostic scores correlate with poor OS in gastric cancer patients. Qiu et al. (26) have demonstrated that the systemic-immune inflammation index is a significant predictor of poor OS but not DFS in gastric cancer. Cao et al. (27) have shown that PLR is also an independent predictor of poor OS in gastric cancer patients.

Another novel marker that has been used in recent years is the SIS. The SIS combines albumin and LMR values and has been noted to be superior to other blood-borne markers like the NLR, modified Glasgow Prognostic Score, and lymphocyte C-reactive protein score for patients with cancer (18, 28). Its prognostic ability has also been validated in several cancer subtypes. Xiong et al. (29) have shown that the SIS can be a simple and useful scoring system to predict OS and DFS in patients with adenocarcinoma of the esophagogastric junction. Feng et al. (12) found the SIS to be an independent predictor of survival and adverse events in patients with rectal cancer. Li et al. (13) have shown that a step-wise increase in SIS significantly shortened OS and DFS in lung-cancer patients undergoing surgical intervention. Similar results have been noted for patients with pancreatic and hepatic cancer as well (30, 31).

Our review, which is the first one to combine data on the prognostic ability of SIS for gastric cancer, concurs with the above-mentioned studies. We noted that SIS was a significant predictor of OS in gastric cancer patients undergoing surgery. Despite including seven studies, we could not combine data in a single meta-analysis due to differences in the reference groups. Our analysis indicated that SIS scores of either 1 or 2 predicted poor OS but the difference in overall HR was not much. The score of 1 vs. O had an HR of 1.25 while 2 vs. 0 had an HR of 1.43. Overall a patient with a high SIS score (1 or 2) had a 36% increased risk of mortality. We also noted that patients with a score of 2 as compared to 0/1 had a higher risk of mortality (HR: 2.53) but data was quite limited. Overall, the study population in the included studies was more or less homogenous in terms of study location, tumor stage, the timing of SIS, and histopathological subtype. All studies were on Asian populations and commonly included stage I–III patients. Most patients had adenocarcinoma and all underwent surgical intervention. Except for one study (19), SIS was measured before surgery in all studies. Also, only one study used the modified version of the SIS. The effects of these singular studies were analyzed using the sensitivity analysis which failed to demonstrate any change in the results. Furthermore, due to a homogenous study population and a limited number of studies in the meta-analysis, we were unable to conduct a subgroup analysis to further decipher the results. Secondly, data on DFS was scarcely reported in the literature with two of three studies noting no association between SIS and DFS. Owing to limited data, strong conclusions cannot be drawn till further studies are published.

Poor prognosis of gastric cancer with high SIS scores could be due to poor patient characteristics and advanced disease stage in such patients. To explore this, we compared the clinicopathological features between high and low SIS scores. Importantly, data was very scarce and the meta-analysis could include just 2–4 studies for each variable. On one hand we noted that larger tumor size correlated with high SIS scores but higher incidence of poorly differentiated tumor was seen in patients with low SIS scores. Also, higher tumor stage did not correlate with SIS scores. Such variability in the results could be primarily due to the scarce data available. Additional data is needed to explore the relationship between gastric cancer features and SIS scores.

The prognostic ability of SIS lies in its combined use of albumin and LMR values. Research indicates SIS is better than individual components for predicting the prognosis (29). The albumin component in SIS indicates the inflammatory as well as the nutritional state of the patient. Hypoalbuminemia is reflective of the presence of malnutrition and cachexia in a cancer patient and is known to be associated with survival outcomes in gastric cancer (32). LMR has two components: lymphocyte and monocyte count. The basic structure of innate and adaptive immunity in an individual is based on lymphocytes which have roles in immune surveillance and immune editing. Lymphocytes have anti-oncogenic potential wherein they limit the growth, invasion, and metastasis of cancer cells (33). The presence of tumor-infiltrating lymphocytes is known to improve the prognosis of cancer patients (34). In contrast to lymphocytes, the presence of monocytes accelerates the growth of tumors by reducing immune surveillance. By means of tumor-monocyte-endothelial interaction, high monocyte counts can increase the risk of metastasis (35, 36). Owing to these postulations, low LMR which is because of low lymphocyte and high monocyte count may lead to poor prognosis in cancer patients.

The primary strength of our review is that it is the first study to pool evidence on the role of SIS in predicting the prognosis of cancer patients. A detailed literature search was undertaken to include maximum studies and a separate analysis of only adjusted outcomes based on reference groups was conducted, thereby presenting high-quality evidence to clinicians. However, there are limitations as well. Our review could include only seven studies all of them retrospective in nature. Retrospective study designs have an inherent bias that cannot be nullified. Study origins only from two countries restrict the generalizability of the results. Also, our inability to perform subgroup analyses due to limited data is another drawback. Lastly, a meta-analysis for DFS could not be conducted, again due to the unavailability of data.

## Conclusions

The SIS score can be a simple and useful tool to predict OS in gastric cancer patients undergoing surgery. Data on DFS is scarce and conflicting. Future studies should report using standard reference groups and provide data on DFS to enhance current evidence.
