# Supplementary material for: Can the systemic inflammation score be used to predict prognosis in gastric cancer patients undergoing surgery? A systematic review and meta-analysis
Source: Front Surg. 2022 Sep 19;9:971326. doi: 10.3389/fsurg.2022.971326 (PMC9633000; doi:10.3389/fsurg.2022.971326)
Supplement: Supplementary file 1 [file Table1.docx]

Supplementary table 1: Search strategy

| **Query** | **Search Details** |
| --- | --- |
| ((cancer) AND (systemic inflammation score)) AND (survival) | ("cancer s"[All Fields] OR "cancerated"[All Fields] OR "canceration"[All Fields] OR "cancerization"[All Fields] OR "cancerized"[All Fields] OR "cancerous"[All Fields] OR "neoplasms"[MeSH Terms] OR "neoplasms"[All Fields] OR "cancer"[All Fields] OR "cancers"[All Fields]) AND (("systemic"[All Fields] OR "systemically"[All Fields] OR "systemics"[All Fields]) AND ("inflammation"[MeSH Terms] OR "inflammation"[All Fields] OR "inflammations"[All Fields] OR "inflammation s"[All Fields]) AND ("score"[All Fields] OR "score s"[All Fields] OR "scored"[All Fields] OR "scores"[All Fields] OR "scoring"[All Fields] OR "scorings"[All Fields])) AND ("mortality"[MeSH Subheading] OR "mortality"[All Fields] OR "survival"[All Fields] OR "survival"[MeSH Terms] OR "survivability"[All Fields] OR "survivable"[All Fields] OR "survivals"[All Fields] OR "survive"[All Fields] OR "survived"[All Fields] OR "survives"[All Fields] OR "surviving"[All Fields]) |
| (((gastric cancer) OR (gastric carcinoma)) OR (gastric malignancy)) AND (systemic inflammation score) | ("stomach neoplasms"[MeSH Terms] OR ("stomach"[All Fields] AND "neoplasms"[All Fields]) OR "stomach neoplasms"[All Fields] OR ("gastric"[All Fields] AND "cancer"[All Fields]) OR "gastric cancer"[All Fields] OR (("gastrics"[All Fields] OR "stomach"[MeSH Terms] OR "stomach"[All Fields] OR "gastric"[All Fields]) AND ("carcinoma"[MeSH Terms] OR "carcinoma"[All Fields] OR "carcinomas"[All Fields] OR "carcinoma s"[All Fields])) OR (("gastrics"[All Fields] OR "stomach"[MeSH Terms] OR "stomach"[All Fields] OR "gastric"[All Fields]) AND ("malign"[All Fields] OR "malignance"[All Fields] OR "malignances"[All Fields] OR "malignant"[All Fields] OR "malignants"[All Fields] OR "malignities"[All Fields] OR "malignity"[All Fields] OR "malignization"[All Fields] OR "malignized"[All Fields] OR "maligns"[All Fields] OR "neoplasms"[MeSH Terms] OR "neoplasms"[All Fields] OR "malignancies"[All Fields] OR "malignancy"[All Fields]))) AND (("systemic"[All Fields] OR "systemically"[All Fields] OR "systemics"[All Fields]) AND ("inflammation"[MeSH Terms] OR "inflammation"[All Fields] OR "inflammations"[All Fields] OR "inflammation s"[All Fields]) AND ("score"[All Fields] OR "score s"[All Fields] OR "scored"[All Fields] OR "scores"[All Fields] OR "scoring"[All Fields] OR "scorings"[All Fields])) |
